# Discovery of a selective and reversible LSD1 inhibitor with potent anticancer effects *in vitro* and *in vivo*

**DOI:** 10.1080/14756366.2025.2466093

**Published:** 2025-02-20

**Authors:** Xiao-Song Zhang, Jin-Zhan Liu, Ying-Ying Mei, Meng Zhang, Li-Wei Sun

**Affiliations:** Xinxiang Central Hospital, The Fourth Clinical College of Xinxiang Medical University, Xinxiang, China

**Keywords:** LSD1, anti-liver cancer effects, H3K4me1/2, migration, epithelial–mesenchymal transition

## Abstract

Lysine-specific demethylase 1 (LSD1) is abnormally overexpressed in various tumour tissues of patients and has been an attractive anticancer target. In this work, a potent LSD1 inhibitor (compound **14**) was designed and synthesised by the molecular hybridisation strategy. It displays the potent antiproliferative activity against HepG2, HEP3B, HUH6, and HUH7 cells with IC_50_ values of 0.93, 2.09, 1.43, and 4.37 μM, respectively. Furthermore, compound **14** is a selective and reversible LSD1 inhibitor with an IC_50_ value of 0.18 μM and increases the methylation levels of H3K4me1/2. Molecular docking studies showed that it formed hydrogen bonds, hydrophilic interactions and hydrophobic interactions with residues of LSD1. Anticancer mechanisms demonstrated that it suppresses migration and epithelial–mesenchymal transition process in HepG2 cells. Importantly, it exhibits potent anti-liver cancer effects *in vivo* without obvious toxic effects. These interesting findings suggested that compound **14**, a novel LSD1 inhibitor, may be a promising therapeutic agent to treat liver cancer.

## Graphical abstract

**Figure:**
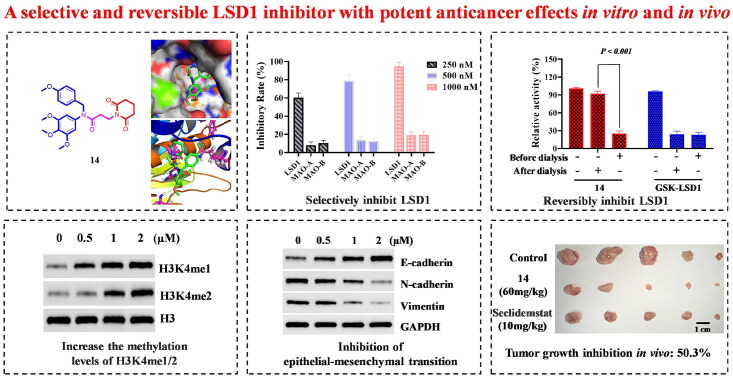

## Introduction

Cancer is a global disease with a high mortality rate, and it imposes an immense threat on the health of human beings around the world^1^. Epigenetics is heritable and dynamic changes in gene functions that occur independently of the DNA sequence variations^2^. Epigenetic modifications can generally be divided into three categories: non-coding RNAs, histone modifications, and DNA or RNA methylations, which are considered to be the main regulatory mechanisms in the progression of cancer^3^. Lysine-specific demethylase 1 ([LSD1], also named KDM1A) as a member of the monoamine oxidase family is the first identified histone demethylase in epigenetics^4^. LSD1 could specifically remove methyl groups from Lys9me1/2 or Lys4me1/2 of histone H3, and it is abnormally overexpressed in various tumour tissues of patients^5^. LSD1 has been an attractive anticancer target, and pharmacological inhibition of LSD1 could significantly slow tumour progression in acute myeloid leukaemia and solid tumours^6^.

**Figure 1.**
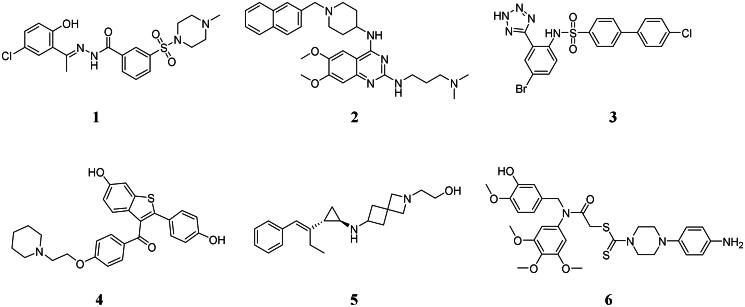
Reported LSD1 inhibitors.

Recently, numerous LSD1 inhibitors with potent anticancer effcts are designed and reported^7^. Seclidemstat (compound **1**, Figure 1) reversibly inhibits LSD1 with an IC_50_ value of 13 nM and has completed the clinical trial to treat patients with advanced solid tumours (NCT03895684)^8^. Quinazoline derivative **2** displays the potent antiproliferative activity with an IC_50_ value of 0.3 μM against THP-1 cells and increases the expression levels of H3K4me2^9^. Tetrazole analogue **3** as a selective LSD1 inhibitor displays the synergistic effect against NB cells with bortezomib^10^. Raloxifene (compound **4**) as a LSD1 inhibitor with an IC_50_ value of 2.08 μM inhibits migration against ACHN cells^11^. Styrenylcyclopropylamine LSD1 inhibitor **5** covalently inhibits LSD1 and shows the potent anticancer effects in a Kasumi-1 xenograft model of acute myeloid leukaemia^12^. *N*-benzylarylamide analogue **6** inhibits LSD1 and induces the accumulation of H3K4me1/2 with potent anticancer effects *in vitro* and *in vivo*^13^. Compared with covalent inhibitors, only a few reversible inhibitors have entered the clinic trials. Therefore, the development of novel and potent reversible LSD1 inhibitors is very significant for the clinical treatment of cancer.

**Figure 2.**
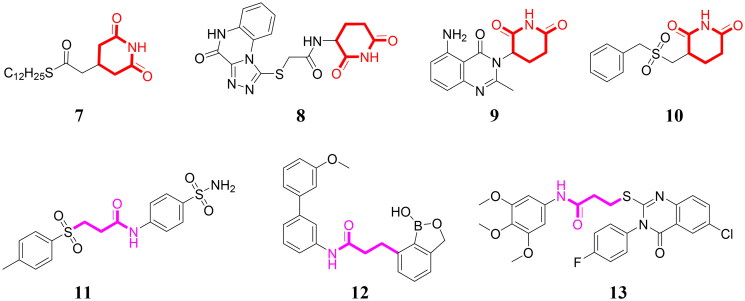
Anticancer agents containing a piperidine-2,6-dione scaffold or a propanamide linker.

Piperidine-2,6-dione scaffold belongs to the privileged fragment in drug design and piperidine-2,6-dione is widely used in the development of anticancer agents. Piperidine-2,6-dione analogue **7** (Figure 2) induces cell cycle arrest and reduces clonogenic capacity in glioblastoma cells^14^. Piperidine-2,6-dione derivative **8** displays the potent antiproliferative activity against HepG2, PC3, and MCF-7 cell lines with IC_50_ values of 9.81, 15.49, and 10.09 μM, respectively^15^. Piperidine-2,6-dione analogue **9** induces apoptosis and inhibits proliferation in diffuse large B-cell lymphoma cells^16^. Piperidine-2,6-dione derivative **10** exhibits the antiproliferative activity with IC_50_ values of 5.52 and 5.17 μM against myeloma MOLP-8 and KMS-12-PE cells^17^. In addition, propanamide scaffold is a promising linker in anticancer agents. Compound **11** with the propanamide linker as a carbonic anhydrase inhibitor displays the antiproliferative activity against HCT-116 and MCF-7 cells with IC_50_ values of 0.57 and 0.67 μM^18^. Compound **12** significantly inhibits colony formation and induces apoptosis against ovarian cancer cells^19^. 4(3*H*)-quinazolinone derivative with the propanamide linker **13** exhibits extensive-spectrum anticancer effects against various cell lines^20^. Thus, piperidine-2,6-dione scaffold and propanamide linker are both promising fragments to design anticancer agents.

**Figure 3.**
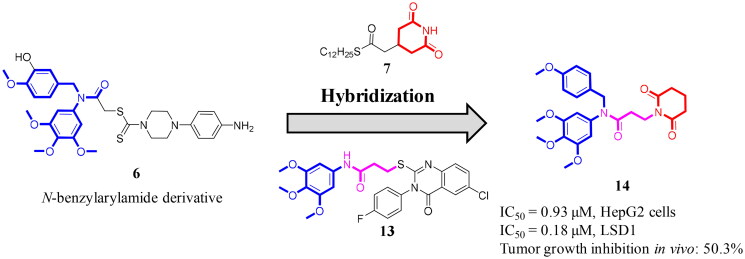
Discovery of a novel LSD1 inhibitor 14 with potent anti-liver cancer effects by the molecular hybridisation strategy.

Molecular hybridisation as a well-exploited drug design strategy could combine different chemical fragments in a novel and bioactive compound^21^. For medicinal chemists, molecular hybridisation has been a powerful tool to discover potent anticancer agents^22^. In this work, targeted compound **14** is designed by the molecular hybridisation strategy based on structures of a *N*-benzylarylamide derivative **6**, a piperidine-2,6-dione analogue **7** and a propanamide derivative **13** (Figure 3). We assumed that linking a piperidine-2,6-dione scaffold and a propanamide linker to the *N*-benzylarylamide moiety could generate compound **14**, which might be a novel LSD1 inhibitor with the potential antiproliferative activity. Luckily, compound **14** was identified as a selective and reversible LSD1 inhibitor with potent anti-liver cancer effects *in vitro* and *in vivo*. To the best of our knowledge, it has not been reported in references regarding a piperidine-2,6-dione-propanamide hybrid as the reversible LSD1 inhibitor with anti-liver cancer effects so far.

**Figure 4.**
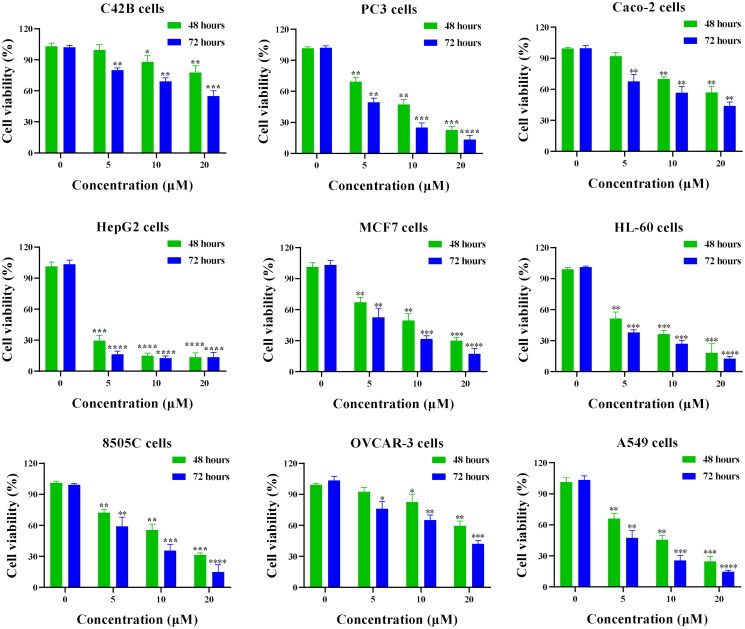
Cell viability of various cancer cell lines treated with compound 14. *p < 0.05, **p < 0.01, ***p < 0.001, and ^****^p < 0.0001.

## Results and discussion

### Synthesis of compound 14

**Scheme 1.**
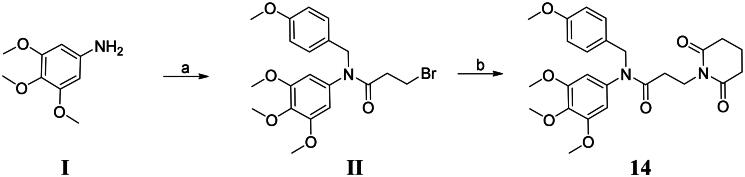
Synthesis of compound **14**. Reagents and conditions: (a) 1-(Chloromethyl)-4-methoxybenzene, potassium carbonate, dichloromethane, 3-bromopropionyl chloride, rt. (b) Piperidine-2,6-dione, triethylamine, acetonitrile, 85 °C.

Following synthetic procedures outlined in Scheme 1, 3,4,5-trimethoxyaniline **I** was reacted with 1-(chloromethyl)-4-methoxybenzene and 3-bromopropionyl chloride in the presence of potassium carbonate at room temperature to obtain the crude intermediate **II** in 83.6% yield. Piperidine-2,6-dione was reacted with intermediate **II** in the presence of triethylamine at 85 °C to obtain the targeted compound **14** in 37.6% yield. Chemical structure of compound **14** was characterised by ^1^H NMR and ^13^C NMR. The detailed data of NMR and HRMS are provided in the Supporting information.

### Compound 14 has a broad-spectrum antiproliferative activity

Human prostate cancer cells (C42B and PC3), colon cancer cells (Caco-2), liver cancer cells (HepG2), breast cancer cells (MCF7), leukaemia cells (HL-60), thyroid cancer cells (8505 C), ovarian cancer cells (OVCAR-3), and lung cancer cells (A549) were cultured to evaluate the viability of compound **14**. 5-Fluorouracil, a broad-spectrum anticancer drug, was selected as the control. In our CCK8 assays, 5-fluorouracil exhibited antiproliferative activities with IC_50_ values of 16.72, 12.28, >20, 9.87, >20, 7.25, 12.37, >20, and >20 μM against C42B, PC3, Caco-2, HepG2, MCF7, HL-60, 8505 C, OVCAR-3, and A549 cell lines, respectively. From results of cell viability in Figure 4, compound **14** could inhibit the proliferation in a concentration-dependent manner against all cell lines. When cancer cells were treated with compound **14** at 10 μM for 48 h, cell viability rates were 87.99%, 52.03%, 72.27%, 14.18%, 49.72%, 35.66%, 49.68%, 80.70%, and 41.07% against C42B, PC3, Caco-2, HepG2, MCF7, HL-60, 8505 C, OVCAR-3, and A549 cells, respectively. All these results indicated that compound **14** has a broad-spectrum antiproliferative activity.

### Compound 14 displays potent anticancer effects in vitro against liver cancer cells

**Figure 5.**
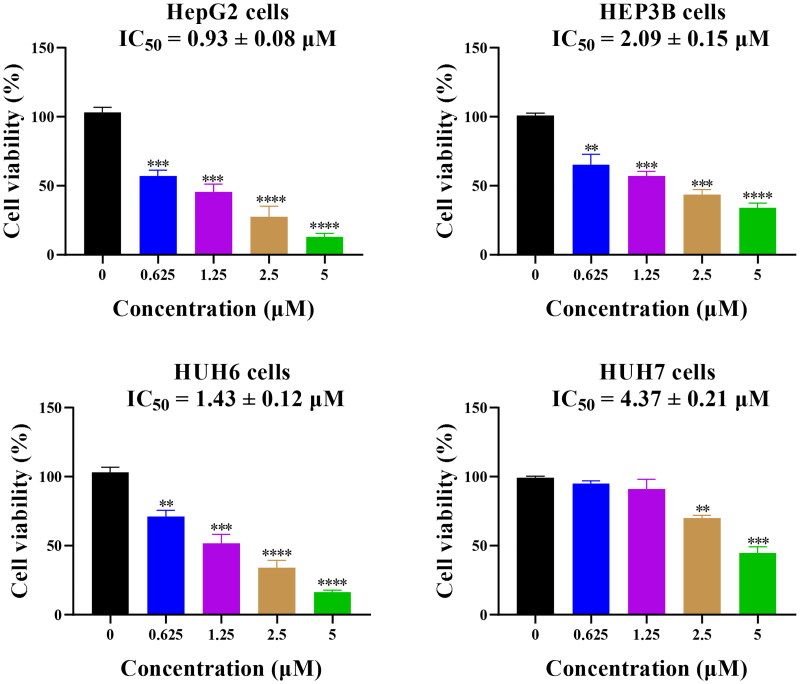
Antiproliferative activity of compound 14 against human liver cancer cell lines. **p < 0.01, ***p < 0.001, and ^****^p < 0.0001.

Among antiproliferative results of above nine cancer cell lines, compound **14** displays the most potent inhibitory activity against liver cancer HepG2 cells. Therefore, four human liver cancer cell lines were selected to evaluate its anticancer effects *in vitro.* Based on the inhibitory results of Figure 5, its IC_50_ values of HepG2, HEP3B, HUH6, and HUH7 cells were 0.93, 2.09, 1.43, and 4.37 μM, respectively. All these results in Figure 5 showed that compound **14** displays potent anticancer effects *in vitro* against liver cancer cells.

**Figure 6.**
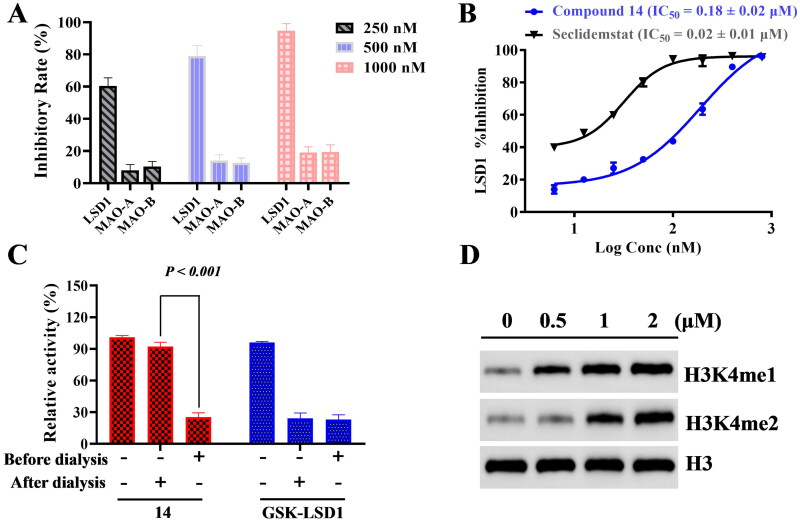
Identification of compound 14 as a potent, selective and reversible LSD1 inhibitor. (A) Inhibitory rates of compound 14 against LSD1, MAO-A, and MAO-B. (B) The inhibitory activity of compound 14 and seclidemstat against LSD1. (C) Dialysis assay to evaluate the reversibility. (D) Western blotting of LSD1 substrates H3K4me1/2.

### Compound 14 is a selective and reversible LSD1 inhibitor

LSD1 has about 70% sequence similarity with monoamine oxidases MAO-A/B^23^. In order to evaluate the selectivity against LSD1, we also tested the inhibitory activity of compound **14** against MAO-A and MAO-B. As shown in Figure 6(A), compound **14** has weak inhibition against MAO-A and MAO-B with IC_50_ values of >1 μM. Seclidemstat as a potent LSD1 inhibitor has entered the clinical trial to treat advanced solid tumours. Thus, seclidemstat was selected as a control drug in the enzymatic activity experiments and animal experiments. Based on the results in Figure 6(B), compound **14** is a potent LSD1 inhibitor with an IC_50_ value of 0.18 μM. An irreversible inhibitor GSK-LSD1 was used in the dialysis assay to evaluate the reversibility of compound **14**. As shown in Figure 6(C), compound **14** inhibits LSD1 in a reversible manner. In addition, compound **14** could increase the methylation levels of H3K4me1 and H3K4me2 as LSD1 substrates in a concentration-dependent manner (Figure 6(D)). All these results indicated that compound **14** is a potent, selective and reversible LSD1 inhibitor.

**Figure 7.**
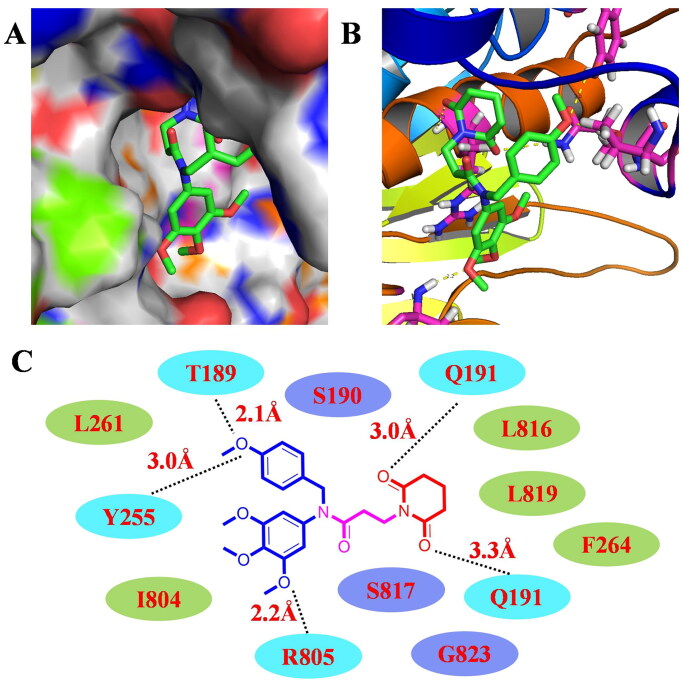
Docking studies of compound 14 targeting LSD1. (A) Surface map of compound 14 in LSD1. (B) Hydrogen bonds between compound 14 and surrounding residues in LSD1. (C) Detailed 2D binding models to show hydrogen bonds, hydrophilic interactions, and hydrophobic interactions.

### Molecular docking studies

3D structure of LSD1 was used as the template for docking studies in this work (PDB: 5LHI). All water molecules, inhibitors and ligands were removed to prepare the PDB file. AutoDock 4.2.6 and PyMOL softwares were used to perform these docking studies. As shown in Figure 7(A,B), compound **14** is located into the active pocket of LSD1. Based on the docking results in Figure 7(C), oxygen atom of 4-methoxybenzyl unit formed two hydrogen bonds with residues T189 (2.1 Å) and Y255 (3.0 Å). Piperidine-2,6-dione scaffold of compound **14** formed two hydrogen bonds with the residue Q191 (3.0 Å, 3.3 Å). 3,4,5-Trimethoxyphenyl unit of compound **14** also formed a hydrogen bond with the residue R805 (2.2 Å). Furthermore, compound **14** formed hydrophilic interactions with residues S190, S817, and G823. It also formed hydrophobic interactions with residues L261, I804, L816, L819, and F264, respectively. These docking results might explain the potent inhibitory activity of compound **14** against LSD1.

**Figure 8.**
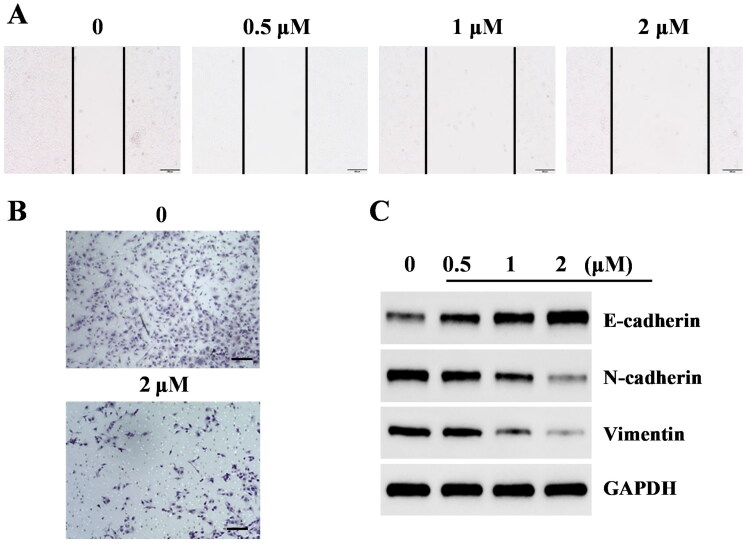
Effects of migration and epithelial–mesenchymal transition process in liver cancer HepG2 cells treated with compound 14. (A) Wound healing assay. (B) Transwell assay. (C) Western blotting analysis of epithelial–mesenchymal transition related proteins.

### Compound 14 inhibits migration and epithelial–mesenchymal transition process in liver cancer HepG2 cells

Recent reference reported that the interruption of LSD1 with siRNA or small molecule inhibitors could inhibit proliferation and migration of cancer cells^24^. Wound healing and transwell assays were performed to investigate migration effects of compound **14** against HepG2 cells. Compared to the migration effect of control group, compound **14** significantly suppresses the migration of HepG2 cells (Figure 8(A,B)). Epithelial–mesenchymal transition plays a crucial role in the occurrence and migration of various tumours. In addition, LSD1 could promote the epithelial–mesenchymal transition in human cancer cells^25^. Western blotting was used to determine expression levels of epithelial–mesenchymal transition-related proteins (E-cadherin, N-cadherin, and Vimentin) in HepG2 cells treated with compound **14**. As shown in Figure 8(C), compound **14** increases the expression level of E-cadherin and decreases the expression levels of N-cadherin and Vimentin in HepG2 cells. All these results investigated that compound **14** inhibits migration and epithelial–mesenchymal transition process in liver cancer HepG2 cells.

**Figure 9.**
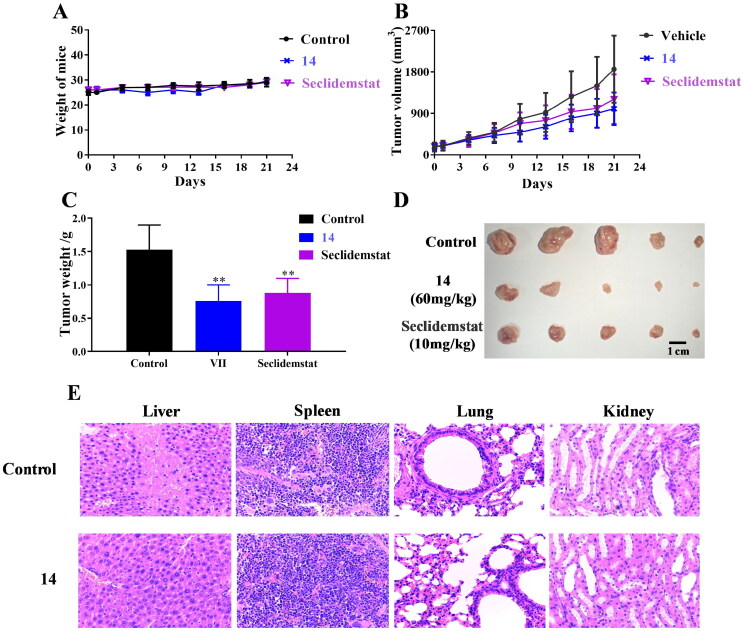
Xenograft studies of compound **14**. (A) Weight of mice. (B) Tumour volume. (C) Tumour weight. (D) The images of tumours. (E) Haematoxylin and eosin staining of liver, spleen, lung, and kidney. **p < 0.01.

### Compound 14 exhibits potent anti-liver cancer effects in vivo

Finally, we subcutaneously inoculated human liver cancer HepG2 cells in nude mice to evaluate anti-liver cancer effects *in vivo* of compound **14**. As shown in Figure 9(A), nude mice treated with compound **14** did not show significant changes in body weight compared to the control group. Furthermore, compound **14** and seclidemstat inhibited the growth of subcutaneous tumours in nude mice (Figure 9(B)). The tumour volume of nude mice treated with compound **14** was significantly lower than that in control group. After the intragastric administration for 21 d, tumours were isolated from nude mice. Tumour weight of nude mice treated with compound **14** was lower than that of control group, and the tumour growth inhibition rate of compound **14** was 50.3% (Figure 9(C)). Haematoxylin and eosin staining of liver, spleen, lung, and kidney in nude mice showed no morphological differences in nude mice treated with compound **14** compared to the control group (Figure 9(D)). These results in animal studies indicated that compound **14** potently inhibits tumour growth in nude mice bearing HepG2 cells without obvious toxic effects.

## Conclusions

In summary, we designed and synthesised a piperidine-2,6-dione-propanamide-trimethoxyphenyl hybrid **14** using the molecular hybridisation strategy. Its potential inhibitory effects against C42B, PC3, Caco-2, HepG2, MCF7, HL-60, 8505 C, OVCAR-3, and A549 cells indicated that compound **14** has a broad-spectrum antiproliferative activity. Compound **14** exhibits potent antiproliferative activity against human liver cancer HepG2, HEP3B, HUH6, and HUH7 cells with IC_50_ values of 0.93, 2.09, 1.43, and 4.37 μM, respectively. Furthermore, compound **14** is a potent, selective and reversible LSD1 inhibitor with an IC_50_ value of 0.18 μM and increases methylation levels of H3K4me1 and H3K4me2. Molecular docking studies showed that compound **14** formed five hydrogen bonds with residues T189, Y255, Q191, and R805 of LSD1. Anticancer mechanisms in liver cancer HepG2 cells investigated that compound **14** inhibits migration and epithelial–mesenchymal transition process. Importantly, compound **14** exhibits potent anti-liver cancer effects *in vivo*. Therefore, compound **14** could be a promising lead compound to discover more potent anticancer agents, and the development of LSD1 inhibitors might be an effective strategy to treat liver cancer.

## Experimental section

### General

PC3 prostate cancer cells, Caco-2 colon cancer cells, HepG2, HEP3B, HUH6, and HUH7 liver cancer cells, MCF7 breast cancer cells, HL-60 leukaemia cells, OVCAR-3 ovarian cancer cells, and A549 lung cancer cells were obtained from Wuhan Pricella Biotechnology Co., Ltd (Wuhan, China). The catalogue numbers of PC3, Caco-2, HepG2, MCF7, HL-60, OVCAR-3, A549, HEP3B, HUH6, and HUH7 cell lines in Wuhan Pricella Biotechnology Co., Ltd are CL-0185, CL-0050, CL-0103, CL-0149, CL-0110, CL-0178, CL-0016, CL-0102, CL-0119, and CL-0120, respectively. C42B prostate cancer cells were obtained from Wuhan SUNNCELL Biotech Co., Ltd (Wuhan, China). The catalogue number of C42B cells in Wuhan SUNNCELL Biotech Co., Ltd is SNL-161. 8505 C thyroid cancer cells were obtained from Shanghai Xuanya Biotechnology Co., Ltd (Shanghai, China). The catalogue number of 8505 C cells in Shanghai Xuanya Biotechnology Co., Ltd is XY-XB-2153. Cell lines were maintained in different medium with 10% foetal bovine serum and 1% penicillin/streptomycin (Servicebio, Wuhan, China). All cell lines were maintained in a humidified atmosphere of 5% CO_2_ at 37 °C. Chemical agents 3,4,5-trimethoxyaniline (**I**), 1-(chloromethyl)-4-methoxybenzene, potassium carbonate, dichloromethane, 3-bromopropionyl chloride, piperidine-2,6-dione, triethylamine and acetonitrile to synthesise compound **14** were obtained from Innochem (Beijing, China).

### Procedure for the synthesis of compound 14

A mixture of 3,4,5-trimethoxyaniline **I** (3 mmol), 1-(chloromethyl)-4-methoxybenzene (3 mmol), potassium carbonate (3 mmol), and dichloromethane (20 ml) was stirred at room temperature for 6 h. Then, 3-bromopropionyl chloride (4 mmol) dissolved in dichloromethane (6 ml) was added. The system was stirred at room temperature for 3 h. The reaction system was filtered and concentrated to obtain the crude intermediate **II**. Yield, 83.6%.

To a stirred solution of piperidine-2,6-dione (4 mmol) and triethylamine (4 mmol) in acetonitrile (10 ml), a solution of the crude intermediate **II** (3 mmol) in acetonitrile (15 ml) was added. The reaction mixture was refluxed at 85 °C for 6 h to synthesise compound **14**. Then, it was purified by column chromatography (petroleum:ethyl acetate = 10:1).

#### 3-(2,6-dioxopiperidin-1-yl)-N-(4-methoxybenzyl)-N-(3,4,5-trimethoxyphenyl)propanamide (14):

Yield: 37.6%, yellow liquid.^1^H NMR (400 MHz, CDCl_3_) δ 7.05 (d, *J* = 8.6 Hz, 2H), 6.73 (d, *J* = 8.6 Hz, 2H), 6.14 (s, 2H), 4.65 (s, 2H), 4.04 − 3.87 (m, 2H), 3.78 (s, 3H), 3.71 (s, 3H), 3.65 (s, 6H), 2.55 (t, *J* = 6.5 Hz, 4H), 2.30 − 2.22 (m, 2H), 1.90 − 1.82 (m, 2H).^13^C NMR (100 MHz, CDCl_3_) δ 171.48, 169.10, 157.96, 152.45, 136.50, 129.52, 128.75, 112.60, 104.81, 59.91, 55.14, 54.26, 51.12, 35.27, 31.78, 30.96, 15.97. HRMS (ESI): [M-H]^-^ calcd. for C_25_H_30_N_2_O_7_: 469.19693, found: 469.19836.

### CCK8 assay

C42B, PC3, Caco-2, HepG2, MCF7, HL-60, 8505 C, OVCAR-3, and A549 were cultured to screen the broad-spectrum antiproliferative activity of compound **14**. For above cell lines, three thousand cells per well were seeded in the 96-well plate and treated by compound **14** for 48 or 72 h at different concentrations (0, 5, 10, and 20 μM). In addition, HepG2, HEP3B, HUH6, and HUH7 were cultured to screen its antiproliferative activity against liver cancer cell lines. For these liver cancer cell lines, two thousand cells per well were seeded in the 96-well plate and treated by compound **14** for 48 h at different concentrations (0, 0.625, 1.25, 2.5, and 5 μM). Then, 10 μl of CCK8 was added into each well and cells were incubated in a humidified atmosphere of 5% CO_2_ at 37 °C for 4 h. The 96-well plate was measured with a microplate reader (Bio-Tek, San Diego, CA) at a wavelength of 450 nm to calculate IC_50_ values.

### Inhibitory activity against LSD1, MAO-A and MAO-B

LSD1 screening assay kit was purchased from Cayman Chemical (Ann Arbor, MI) to evaluate the enzymatic activity of compound **14**. An aliquot of 20 μl of LSD1 assay reagent, 20 μl LSD1 assay peptide, 120 μl of LSD1 buffer solution and 10 μl of compound **14** at different concentrations (6.25, 12.5, 25, 50, 100, 200, 400, 800, and 1000 nM) were added to each well in the 96-well plate. After the incubation for 30 min at 37 °C, a mixture of horseradish peroxidase solution (20 μl) and fluorometric substrate solution (10 μl) was added to each well. Then, the 96-well plate was incubated for 15 min at 37 °C. Finally, the fluorescence of each well in the 96-well plate was measured at an emission wavelength of 590 nm and an excitation wavelength of 530 nm by a fluorimeter (Agilent Technologies Inc., Palo Alto, CA). Seclidemstat, a famous LSD1 inhibitor, was used as the control drug in this assay.

In order to analyse the enzymatic selectivity of compound **14**, MAO-A/B was purchased from Servicebio (Wuhan, China). MAO-A was dissolved in phosphate buffer (0.1 M, pH = 7.4) to get MAO-A enzymatic buffer with a concentration of 0.006 mg/ml. MAO-B was also dissolved in phosphate buffer (0.1 M, pH = 7.3) to get MAO-B enzymatic buffer with a concentration of 0.015 mg/mL. Kynuramine dihydrochloride (Servicebio, Wuhan, China) was used to get the assay buffer with a concentration of 40 μM for MAO-A and 20 μM for MAO-B. An aliquot of 97 μl of assay buffer and 3 μl of compound **14** at different concentrations (250, 500, and 1000 nM) were added into each well on the 96-well black plate. The plate was incubated at 37 °C for 20 min, and 80 μl of 2 N sodium hydroxide was added into each well to stop the reaction. The fluorescence of each well was measured using a microplate reader (Agilent Technologies Inc., CA) with an excitation at 310 nm and an emission at 380 nm.

### Dialysis assay

A mixture of compound **14** and the recombinant LSD1 was incubated for 60 min at 37 °C. Then, the system was dialysed against 50 mmol/l HEPES buffer at 37 °C for 12 h. Finally, the HEPES buffer was replaced and the system was incubated for 12 h at 37 °C. In the dialysis experiment, an irreversible inhibitor GSK-LSD1 was purchased as the reference drug (Innochem, Beijing, China).

### Western blotting

Compound **14** at different concentrations (0, 0.5, 1, and 2 μM) was added into cancer cells solution and incubated for 48 h. Total protein was isolated from cells using RIPA buffer (Servicebio, Wuhan, China). An amount of 30 mg of total protein were separated by sodium dodecyl sulphate polycrylamide gel electrophoresis and transferred to PVDF membranes. 5% non-fat milk (Servicebio, Wuhan, China) was used to block PVDF membranes and incubated with primary antibody for 12 h at 4 °C. Then, PVDF membranes were washed by TBST buffer (Servicebio, Wuhan, China) and incubated with secondary antibody at room temperature for 2 h. The enhanced chemiluminescence kit (Servicebio, Wuhan, China) was used to visualise immunoreactive bands. H3, GAPDH, Vimentin, N-cadherin, and E-cadherin as antibodies were purchased from Servicebio (Wuhan, China). Catalogue numbers of H3, GAPDH, Vimentin, N-cadherin, and E-cadherin as antibodies in Servicebio are GB11102, GB15004, GB11192, GB12135, and GB12868, respectively. H3K4me1 and H3K4me2 were obtained from Proteintech Group, Inc (Wuhan, China). Catalogue numbers of H3K4me1 and H3K4me2 as antibodies in Proteintech Group are 91289 and 39679, respectively.

### Molecular docking

Protein structure of LSD1 was obtained from protein data bank (https://www.rcsb.org/). The resolution of LSD1 in molecular docking studies was 3.40 Å (PDB code: 5LHI). Water and ligand molecules (FAD, 6 × 5 and GOL) of the protein were deleted. AutoDock 4.2.6 was used to add hydrogen and partial charges. In the docking process, number of points in x-dimension, y-dimension and z-dimension were 70, 50, and 86, respectively. Docking results between compound **14** and LSD1 were reserved as a PBD file. Hydrogen–bond interaction, hydrophobic interaction and hydrophilic interaction were studied by PyMOL.

### Wound healing

Cancer cells were seeded into a 6-well plate for 12 h and each well was scratched using a sterile micropipette tip. Cellular debris was removed by PBS solution (Servicebio, Wuhan, China), and compound **14** at different concentrations (0, 0.5 μM, 1 μM, and 2 μM) was added. The 6-well plate was incubated for 48 h and photographed on a microscope.

### Migration

Cancer cells were seeded into a Transwell 24-well plate (Corning, NY) and compound **14** was added. About 20% foetal bovine serum and 1% heat-inactivated foetal bovine serum were respectively added into the lower and upper chamber. The 24-well plate was incubated for 48 h at 37 °C, and it was washed by water. Cancer cells were fixed with cold ethanol for 20 min and stained with haematoxylin solution (Servicebio, Wuhan, China).

### Xenograft study

Tumorigenesis in nude mice could stimulate the growth process of human tumours in the body, and could be used to develop and evaluate the effectiveness and safety of anti-cancer candidates. Therefore, xenograft studies in nude mice were performed to evaluate anticancer effects of compound **14**. Animals were treated according to the protocols established by the ethics committee of The Fourth Clinical College of Xinxiang Medical University. The *in vivo* experiments were carried out in accordance with the approved guidelines and approved by the ethics committee of The Fourth Clinical College of Xinxiang Medical University. The number of ethics approvals is XMU-2024–01-03. All authors have adhered to the ARRIVE guidelines (https://arriveguidelines.org/). BALB/c nude mice were purchased from Beijing Huafukang Biotechnology Co., LTD (Beijing, China), and fifteen mice were used in this study (aged 6–7 weeks and weighing 20–30 g). Before the animal experiments, all nude mice were given a week to adjust to the clean, hygienic and specific-pathogen-free environment. All nude mice were taken care of instainless steel cages with a constant temperature of 60% humidity, 25 °C and a 12/12 light/dark cycle. Nude mice were fed with refreshing water and nourishing food everyday.

Then, nude mice were anaesthetised with 2% isoflurane and 0.3 l/min of oxygen by the small animal anaesthesia machine (Beijing Yi Zejia Technology Co., LTD, Beijing, China). 3 × 10^5^ human liver cancer HepG2 cells were subcutaneously injected in the right armpit of mice. Until the volume of tumours reached 60–100 mm^3^, all mice were randomly divided into three groups and each group had five nude mice. An amount of 60 mg/kg of compound **14**, 10 mg/kg of seclidemstat and water as the control group were given by gavage every day for 21 days. Weight of mice and tumour volume were measured every 3 d. All nude mice in this xenograft studies were performed euthanizing procedures using a carbon dioxide euthanasia chamber (Shanghai Yuyan Instruments Co., Ltd, Shanghai, China). The flow rate of carbon dioxide was adjusted at 20% of the volume of the euthanasia chamber per minute. Once nude mice were unconscious, the flow rate was increased to 100% of the euthanasia chamber volume per minute. Death was confirmed by immobility, non-breathing and pupil enlargement. Tumours form BALB/c nude mice were isolated and weighed.

### Haematoxylin and eosin staining

Liver, spleen, lung, and kidney were isolated from BALB/c nude mice and stained by haematoxylin and eosin solution. The detailed methods were performed according to a reported reference^26^. First, liver, spleen, lung, and kidney were added into tubes of 4% paraformaldehyde fixative (Servicebio, Wuhan, China). Then, tissues were dehydrated in the alcohol solution, and xylene was added into the solution for 2 h. Finally, sectioning and staining were performed to finish this experiments.

### Statistical analyses

All biological data were obtained at least three independent experiments. Statistical differences of two groups were determined with analysis of Student’s *t*-test. *p < 0.05, **p < 0.01, ***p < 0.001, and ^****^p < 0.0001 were considered statistically significant.

## Supplementary Material

A statement for the xenograft study.doc

## Data Availability

The data in this study are available from the corresponding author upon the reasonable request.
