# Supplementary material for: Discovery of a selective and reversible LSD1 inhibitor with potent anticancer effects in vitro and in vivo
Source: J Enzyme Inhib Med Chem. 2025 Feb 20;40(1):2466093. doi: 10.1080/14756366.2025.2466093 (PMC11843658; doi:10.1080/14756366.2025.2466093)
Supplement: A statement for the xenograft study.doc [file IENZ_A_2466093_SM6257.doc]

**A statement for the xenograft study**
***- The name of the ethics committee or institutional review board that approved the study***

The name of the ethics committee was the ethics committee of The Fourth Clinical College of Xinxiang Medical University.

***- The number or ID of the ethics approval***

The number of ethics approvals is XMU-2024-01-03.

***- Justification for use and number of animals***

Tumorigenesis in nude mice could simulate the growth process of human tumors in the body, and could be used to develop and evaluate the effectiveness and safety of anti-cancer candidates. Therefore, xenograft studies in nude mice were performed to evaluate anticancer effects of compound **14**. Fifteen BALB/c nude mice were used in this study (aged 6-7 weeks and weighing 20-30 g).

***- Information on housing, feeding, and environmental enrichment***

All nude mice were given a week to adjust to the clean, hygienic and specific-pathogen-free environment. All nude mice were taken care of instainless steel cages with a constant temperature of 60% humidity, 25°C and a 12/12 light/dark cycle. Nude mice were fed with refreshing water and nourishing food everyday.

***- Information on mode of analgesia and anaesthesia and steps taken to minimize suffering***

Nude mice were anaesthetised with 2% isoflurane and 0.3 L/min of oxygen by the small animal anesthesia machine (Beijing Yi Zejia Technology Co., LTD, Beijing, China).

***- Mode of sacrificing the animals***

All nude mice in this xenograft studies were performed euthanizing procedures using a carbon dioxide euthanasia chamber (Shanghai Yuyan Instruments Co., Ltd, Shanghai, China). The flow rate of carbon dioxide was adjusted at 20% of the volume of the euthanasia chamber per minute. Once nude mice were unconscious, the flow rate was increased to 100% of the euthanasia chamber volume per minute. Death was confirmed by immobility, non-breathing and pupil enlargement.

***- A declaration that the authors have adhered to the ARRIVE guidelines (***[***https://arriveguidelines.org/***](https://arriveguidelines.org/)***)***

All authors have adhered to the ARRIVE guidelines (<https://arriveguidelines.org/>).
